# Supplementary material for: SNX17 Recruits USP9X to Antagonize MIB1-Mediated Ubiquitination and Degradation of PCM1 during Serum-Starvation-Induced Ciliogenesis
Source: Cells. 2019 Oct 29;8(11):1335. doi: 10.3390/cells8111335 (PMC6912348; doi:10.3390/cells8111335)
Supplement: Supplementary file 1 [file cells-08-01335-s001.pdf]

Supplementary Materials

Supplementary Methods

Quantitative RT-PCR. Total RNA was extracted from RPE1 cells using Trizol (Thermo Scientific) in accordance with the manufacturer's protocol. cDNA was synthesized with the ReverTra Ace qPCR RT Kit (FSQ-101, TOYOBO) and quantitative PCRs were performed using the BIO-RAD CFX96 real time system according to the manufacturer's instructions (BIO-RAD). GAPDH was used as the internal control. The sequences of PCR primers used in were: GAPDH (5′-GGTCGGAGTCAACGGATTTG-3′, 5′-GGAAGATGGTGATGGGATTTC-3′); PCM1 (5′-TCCTTGCCAGAGATCCTCA-3′, 5′-CTACCTGCAGTTTCTGCAACA-3′); CEP131 (5′-TTCCAGTGCCCTTGACTCAC-3′, 5′-GATGTTGTTGAGGGAGGGGG-3′); OFD1 (5′-AGGCGCCTCTAACTCTTTAGT-3′, 5′-CAGATCCTGAAACCAGTGATTTGT-3′).

Transmission electron microscopy (TEM) assay. WT and SNX17 knockout RPE1 cells were cultured in serum-free DMEM-F12 media for 24 h, fixed with 2.5% glutaraldehyde at 4 °C overnight, washed with PBS then post-fixed in 2% OsO_4 for 90 min. Cells were dehydrated using ascending ethanol concentrations and propylene oxide, embedded in Epon812 (02660, SPI) and polymerized at 60 °C for 48 h. Samples were then mounted on aluminum stubs, and serial ultrathin sections (100 nm) were prepared with the ultramicrotome (EM UC7, Leica) and stained with uranyl acetate and lead citrate. Samples were then examined and photographed using the Tecnai G2 Spirit TEM (FEI).

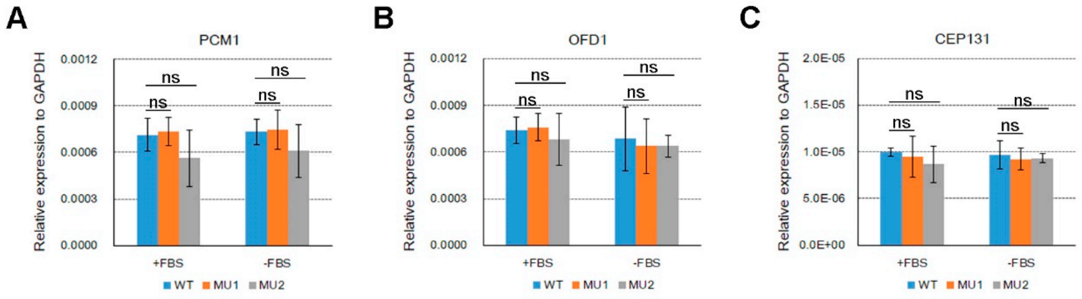

Figure S1. qRT-PCR analysis for (**A**) pericentriolar material 1 (PCM1), (**B**) OFD1, and (**C**) CEP131 gene expression in WT and SNX17 knockout RPE1 cells. Data represent mean ± SD from three independent biological repeats. Note: ns, non-significant in one-way ANOVA with Tukey’s multiple comparison test.

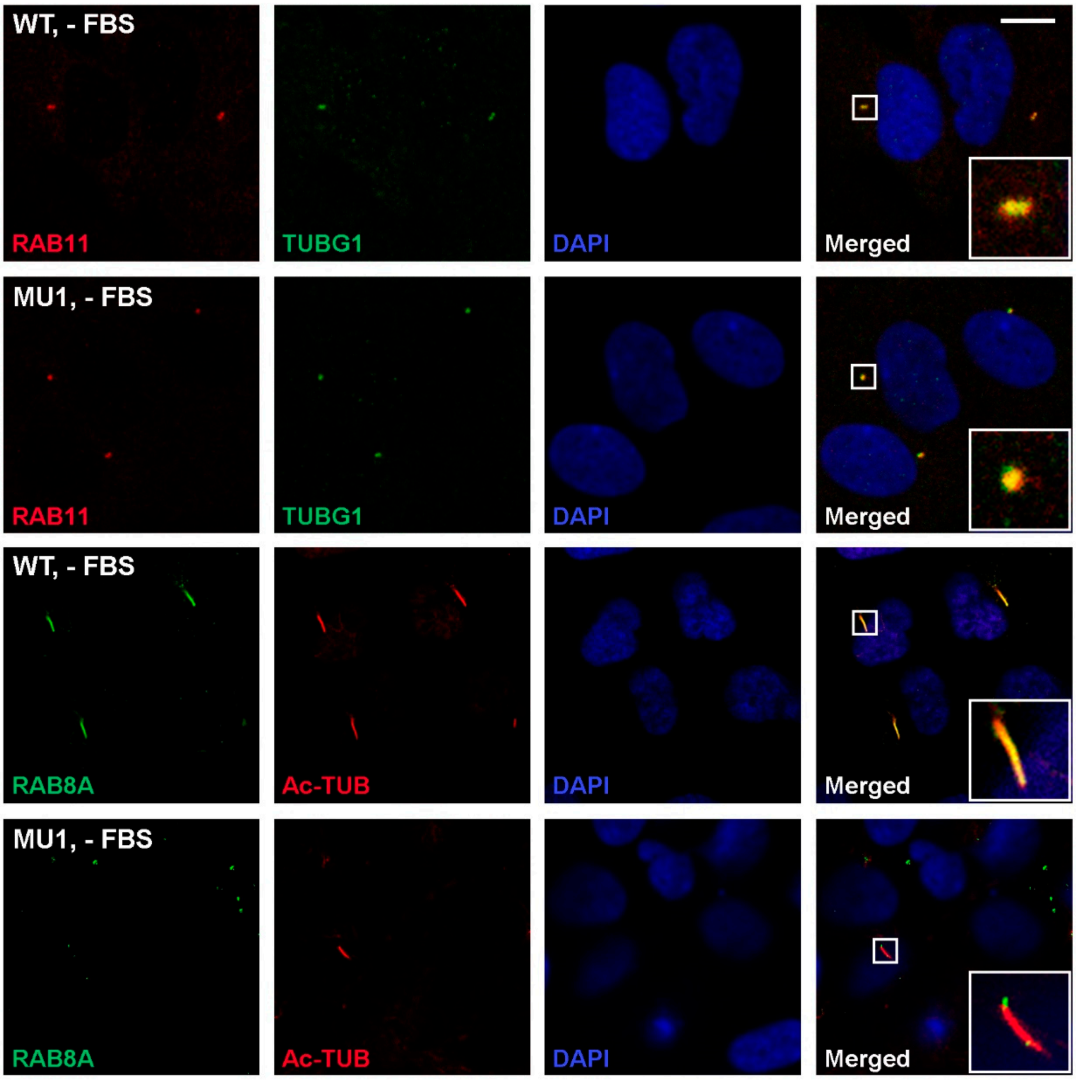

Figure S2. Immunofluorescence staining for intracellular distribution of RAB11 or RAB8A in WT and SNX17 mutant RPE1 cells. Cells were serum-starved for 48 h and stained for endogenous RAB11, RAB8A, and TUBG1. Co-localization of RAB11 with TUBG1 were detected in both WT cells (38/38) and MU1 cells (42/42). For ciliary localization of RAB8A, most of the cilia in WT cells were RAB8A-positive (30/33) while 8 out of 9 cilia in MU1 were RAB8A-negative. Scale bar: 10 µm.

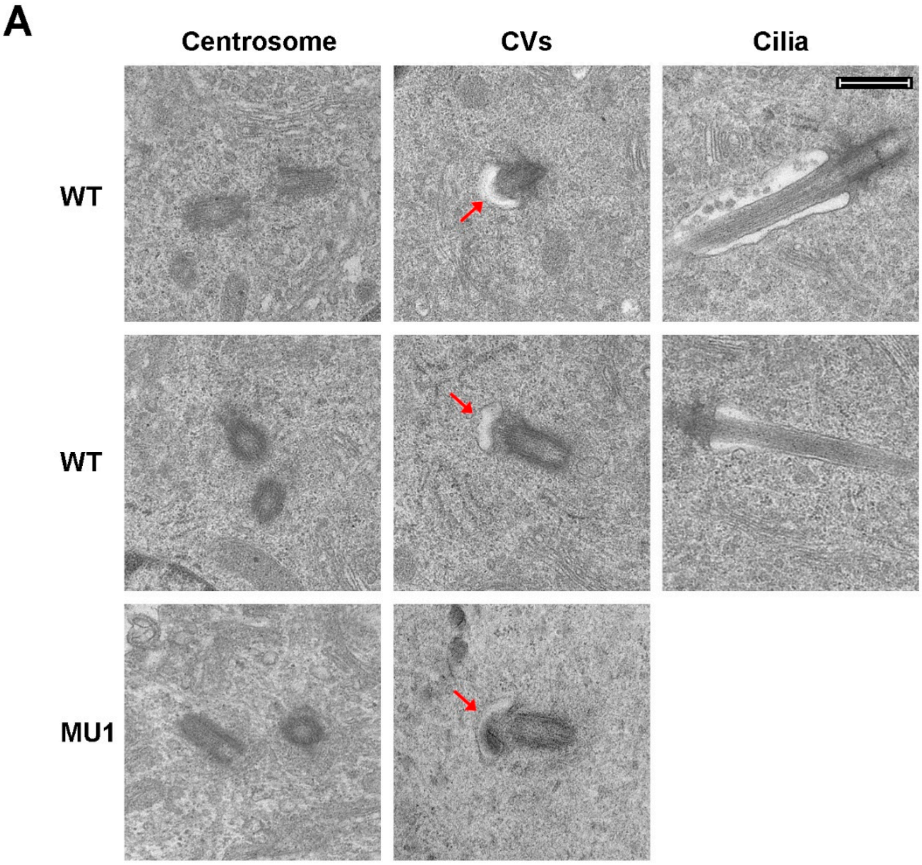

B

Cell type	Centrosome	CVs	Cilium
WT	20	7	3
MU1	25	1	0

Figure S3. Transmission electron microscopy (TEM) analysis of centrosome and ciliary vesicles (CVs) in WT and SNX17 mutant cells. (**A**) Representative TEM images at 24 h after serum starvation. Samples were grouped into three categories: centrosome without ciliary vesicles (Centrosome), centriole with ciliary vesicles (CVs, arrows in **A**), or cilia. Scale bar: 500 nm. (**B**) Statistical results for (**A**). Three sets of samples for WT and MU1 were analyzed and the sum numbers of each category were listed in the table.

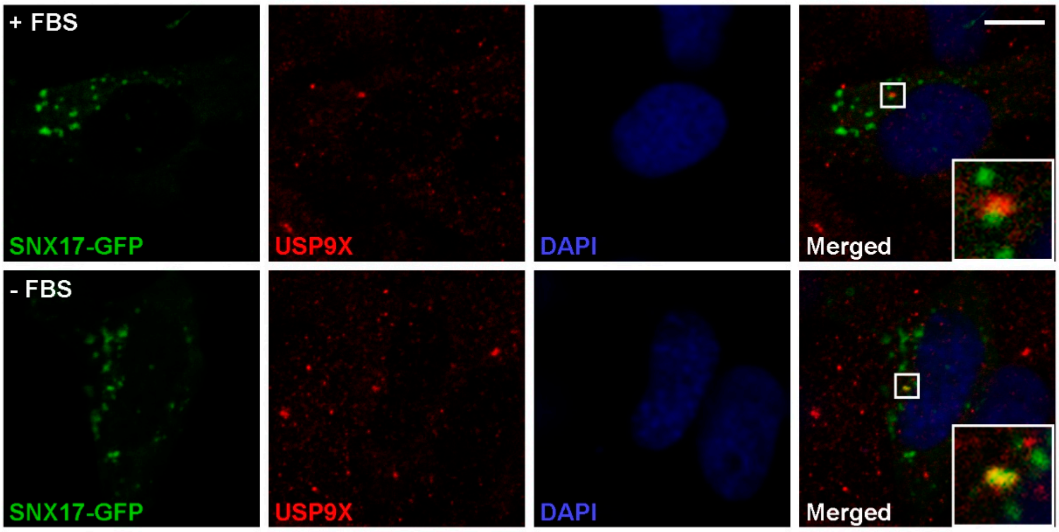

Figure S4. Subcellular distribution of SNX17 and USP9X in RPE1 cells. Cells were transfected with SNX17-GFP, grown in either serum-containing media or serum-free media for 24 h, and then fixed for immunofluorescence staining. Co-localization of SNX17-GFP with USP9X were detected in the presence of serum (4 out of 7 cells examined) or absence of serum (5 out 6 cells examined). Scale bar, 10 μm.

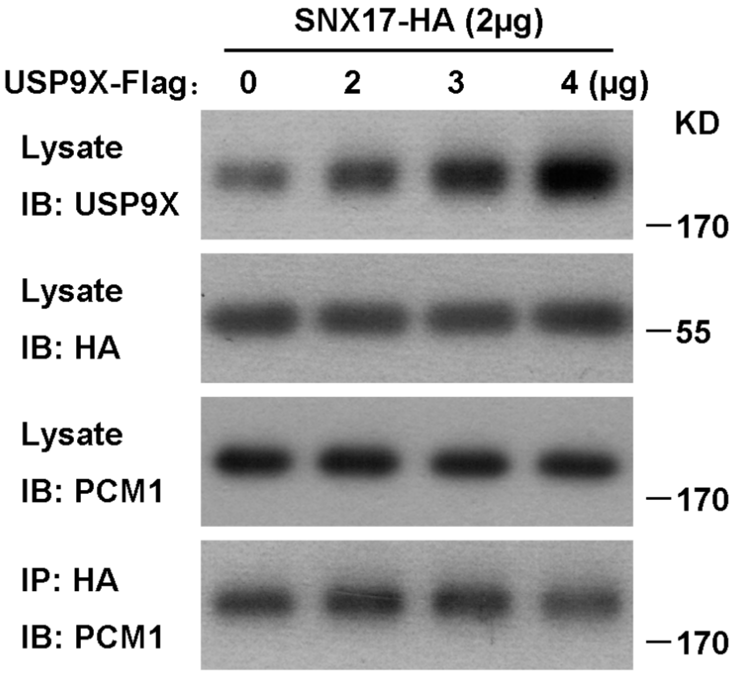

Figure S5. SNX17 pull-down of PCM1 in variable amounts of USP9X. Cells were co-transfected with SNX17-HA and the indicated amounts of USP9X-Flag for 48 h. Cells were then harvested and immunoprecipitated with HA-tag, and the protein level of PCM1 in immune-complex was determined by western blot. The left lane in USP9X blot indicates the endogenous USP9X protein level. Increasing USP9X protein level by ectopical expression had little effect on co-immunoprecipitation of SNX17 and PCM1.

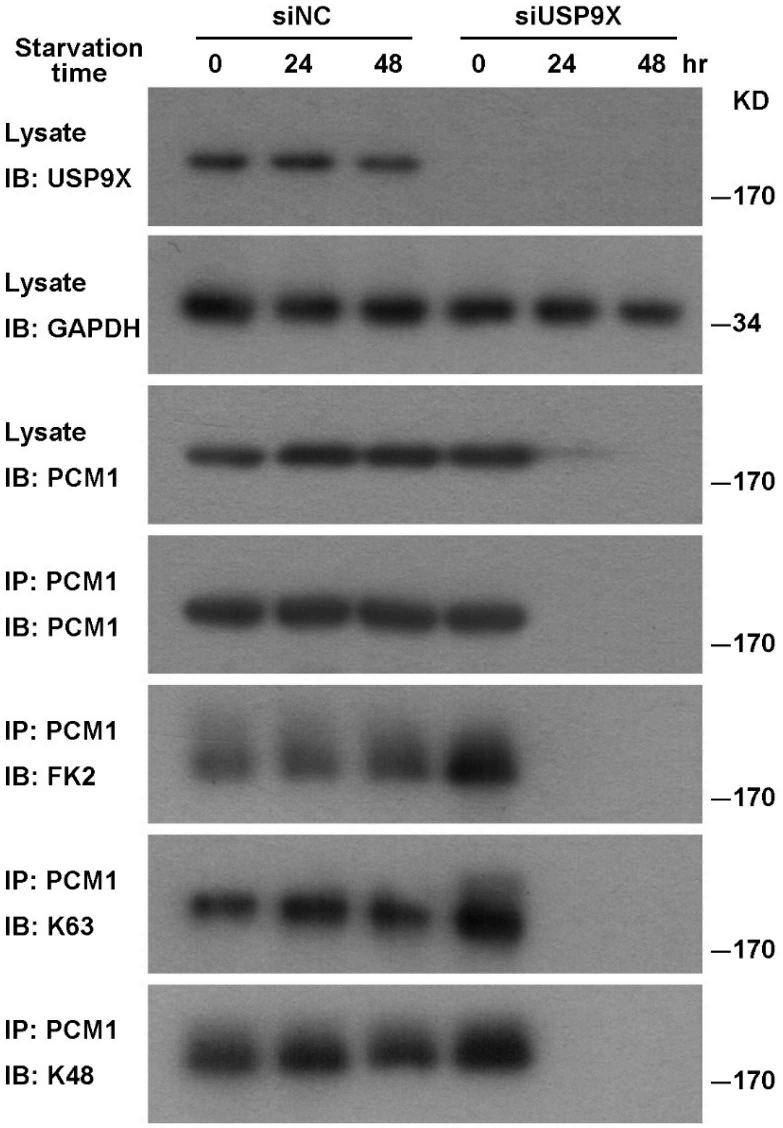

Figure S6. Analysis of PCM1 ubiquitination upon USP9X knockdown. Cells were transfected with a pool of siRNAs to USP9X or a control siRNA for 24 h, and then serum-starved for the indicated times. Cells were then harvested for immunoprecipitation and western blot. Twenty four hours of siUSP9X treatment in the serum media did not induce the degradation of PCM1; however, PCM1 in these cells were rapidly degraded upon serum starvation. Total ubiquitination as well as the K63-linked ubiquitination were elevated upon siUSP9X treatment.

Table S1. List of antibodies used in this study.

Name of antibody	Company	Catalog #	Dilution (IF)	Dilution (WB)
BBS4 (Rabbit)	Proteintech	12766-1-AP		1:500
CEP97 (Rabbit)	Sigma	HPA002980		1:1000
CEP131 (Rabbit)	Sigma	HPA024019		1:200
CP110 (Rabbit)	Proteintech	12780-1-AP		1:500
FLAG (Rabbit)	Sigma	F7425		1:5000
GAPDH (Mouse)	Abcam	ab9482		1:3000
LAMP2 (Mouse)	Abcam	ab25631	1:500	
MIB1 (Rabbit)	Abcam	ab124929	1:200	1:400
OFD1 (Rabbit)	Sigma	SAB2702042		1:500
PCM1 (Mouse)	Santa cruz	sc-398365	1:500	
PCM1 (Rabbit)	CST	5213	1:500	1:500
Pericentrin (Rabbit)	Abcam	ab4448	1:2000	
Pericentrin (Rabbit)	millipore	ABT59		1:1000
RAB8A (Goat)	Abcam	ab77624	1:1000	
RAB8A (Rabbit)	Abcam	ab188574		1:1000
RAB11 (Rabbit)	CST	5589	1:1000	1:1000
SNX17 (Mouse)	Santa cruz	sc-166585		1:400
USP9X (Rabbit)	Proteintech	55054-1-AP	1:200	1:500
Acetylated Tubulin (Mouse)	Sigma	T7451	1:1000	1:1000
Gamma-Tubulin (Mouse)	Sigma	T5326	1:1000	1:1000
Anti-Ubiquitin FK2 (Mouse)	Enzo	BML-PW8810-0500	1:500	1:1000
Anti-Ubiquitin Antibody, Lys63-Specific (Rabbit)	Millipore	05-1308	1:1000	1:1000
Anti-Ubiquitin Antibody, Lys48-Specific (Rabbit)	Millipore	05-1307	1:1000	1:1000

HA antibody (mouse)	Beyotime	AH158		1:1000
Goat anti-Mouse IgG Alexa Fluor 488	Thermo fisher	A-11029	1:500	
Goat anti-Mouse IgG Alexa Fluor 568	Thermo fisher	A-11031	1:500	
Goat anti-Rabbit IgG Alexa Fluor 405	Thermo fisher	A-31556	1:500	
Goat anti-Rabbit IgG Alexa Fluor 488	Thermo fisher	A-11034	1:500	
Goat anti-Rabbit IgG Alexa Fluor 568	Thermo fisher	A-11036	1:500	
Goat Anti-Mouse IgG (HRP)	Abcam	ab6789		1:3000
Goat Anti-Rabbit IgG (HRP)	Abcam	ab6721		1:3000
